# What is special about suicidal depression?

**DOI:** 10.1192/j.eurpsy.2021.103

**Published:** 2021-08-13

**Authors:** P. Courtet, E. Olie

**Affiliations:** 1 Department Of Emergency Psychiatry & Acute Care, University of Montpellier., Montpellier, France; 2 Psychiatric Emergency And Acute Care, CHU Montpellier, montpellier, France

**Keywords:** Suicidal ideation, prospective study, Depression, bipolar disorder

## Abstract

**Objective:**

Bipolar disorder is one of the most frequent psychiatric disorders among suicidal patients. A large part of patients with bipolar disorder (30–50%) will attempt suicide. Suicidal ideation being a major risk factor of suicidal act, it is crucial to better characterize patients with suicidal bipolar depression (i.e. depression with current suicidal ideation). The aim of this study was to characterize suicidal bipolar depressed patients in comparison with non-suicidal depressed patients in terms of clinical characteristics, evolution of depression and suicidal ideation course over time, and risk of suicide attempt during follow-up.

**Methods:**

Among patients with bipolar disorder recruited from the network of FondaMental expert centres for bipolar disorder between 2009 and 2017, we selected patients with at least mild depression and without current manic symptomatology at baseline (N = 938). Suicidal depression was defined by a baseline score ⩾2 for item 12 of the QIDS-SR (28.9%). A subsample of about 300 patients (w/ or w/o suicidal ideation at baseline) was followed up for 2 years.

**Results:**

Baseline clinical features (e.g. depression severity, childhood trauma, global functioning) were more severe in patients with without suicidal depression. Suicidal patients tended to remain more suicidal throughout the followup (3.4-fold higher risk of persistent suicidal ideation at the 2-year visit despite an improvement in depressive symptomatology).

**Conclusions:**

Depressed bipolar disorder patients reporting suicidal ideation had more severe clinical features and were more prone to report persistent suicidal ideation during the follow-up, independently of thymic state. Clinicians should closely monitor this subgroup of patients

**Disclosure:**

No significant relationships.

